# Hospital and Institutional News

**Published:** 1911-11-25

**Authors:** 


### HOSPITAL AND INSTITUTIONAL NEWS.

### COMING MEETING OF THE BRITISH HOSPITALS ASSOCIATION.

Arrangements have been made to hold a meeting of hospital managers, representing the voluntary hospitals in every district in the country, at the Westminster Palace Hotel, London, on Friday, December 1, at 4.30 p.m. Members of Parliament and others interested in the question have intimated their intention of being present. Already most of the principal hospitals, amounting collectively to nearly two hundred, have decided to send representatives to this meeting. It is now assured that this gathering will prove the most representative meeting of hospital managers that has ever been held in this country, and probably in the whole world. The British Hospitals Association has all along maintained that the National Insurance Bill is incomplete unless it provides for the hospital treatment which insured persons must have if their medical needs are to be covered by the Bill. This view has since been accepted by the British Medical Association, which has agreed to press the Government again to make adequate provision for institutional treatment. Thus hospital benefit becomes one of the recommendations of the Council to the Special Representative Meeting to be held on November 23, after we go to press.

### LONDON MENTAL HOSPITAL APPOINTMENTS.

Various changes have lately been made in the staffs of the London mental hospitals. Dr. Geoffrey Clerke, who was recently appointed to be first assistant medical officer at Colney Hatch, has been transferred to fill the vacancy for a first assistant at Banstead Mental Hospital caused by the resignation of Dr. Murphy. Mr. S. C. Elgee, second assistant medical officer at Colney Hatch, has been promoted to be first assistant; and at Long Grove Mental Hospital, Mr. Bernard Hart, third assistant medical officer, has been promoted to be second assistant.

### A MEDICAL OFFICER'S GRIEVANCE.

District Medical Officers will read with interest a summary of the grievance of Dr. Owen Coleman, District Medical Officer for Surbiton, which he sent in a recent lengthy letter to the Kingston Board of Guardians. His complaint was that owing to a departure from the routine methods of carrying out the Lunacy Act of 1890, he and his fellow district medical officers were deprived of their fees for the certification of lunatics of the poorest class. Dr. Coleman quoted two instances to show that by treating all lunacy cases as if they were "urgent," within the meaning of the Act, the work and trouble leading to certification fell on the District Medical Officer, while the guinea was fobbed by the resident officer to the Infirmary. The Chairman of the Guardians, Mr. A. Sawer, in reply urged that it was the responsibility of the magistrate, not of the Guardians, to call in a medical man, and that he alone had power to do it. In the second place, he said, there had been no departure from practice, as the relieving officer was never sent for until the case was "urgent" enough to require removal; and lastly that, in fairness to the patients (to whom the first duty of the Guardians lay) they could not be kept waiting for the daily visits of the medical officer from the district from whence they came, when there was at the infirmary a resident medical officer perfectly competent to sign the certificate. It is expected that a letter embodying these arguments will be presented at the next meeting, and, if approved, sent in reply to Dr. Coleman.

### "THE SCANDALS AT GUY'S."

Under this title Dr. Salter of Bermondsey contributed an interesting article to a recent number of the *Southwark and Bermondsey Recorder*. Dr. Salter is one of the most distinguished Guy's men in London; he is thoroughly acquainted with the administration of the hospital on the one hand, and the shortcomings of the Poor Law system on the other, and his large experience as a general practitioner in the district wherein Guy's is situate gives him an authority to speak on the matter. His conclusions will therefore be read with interest. The main one is that he considers the hospital population of South London far in excess of the accommodation provided by the voluntary institutions in that district, and pleads for State-supported institutions. His paper is a very thoughtful contribution to the discussion concerning the upkeep of the voluntary system, and deserves careful consideration from both supporters and attackers of the system. The current number of the *Guy's Hospital Gazette* (which being a censored organ may be expected to voice the official opinion of the hospital authorities) comments on the article in the following remarks: "While thoroughly agreeing that reform of some kind is needed, and needed urgently, we cannot agree that nationalisation of our hospitals is the solution of the difficulty. In our opinion there is a remedy for hospital overcrowding, less drastic, and also, we think, more practical than the one Dr. Salter suggests. The provision of self-supporting paying hospitals for middle-class patients would greatly relieve the congestion of the free hospitals, and at the same time prevent a good deal of abuse of charity, while if the homes of the poor were a little less like pigsties and a little more in accordance with the elements of hygiene, a good many patients could be treated at home without ever having to go to a hospital at all."

### CHESTERFIELD'S NEW MEDICAL OFFICER.

On December 1 Dr. Frederick A. Sharpe, late resident medical officer of the Derby Isolation Hospital, and of the Phthisis Sanatorium, takes up his appointment as Medical Officer to Chesterfield. Dr. Sharpe, who is an M.D. of London University, State Medicine, a Guy's man, and holds the Leeds Diploma of Public Health, was educated at Chesterfield Grammar School in the earlier days of its present headmaster, Mr. J. Mansell. After qualifying in 1905, he went to York as surgeon to the dispensary till July 1907. From there he removed to Manchester, where he was appointed one of the resident medical officers of the Monsall Fever Hospital, to which he finally became deputy superintendent. Dr. Sharpe is thirty years old, and a bachelor.

### THE EDINBURGH ANTI-PHTHISIS SCHEME.

We hear a lot of campaigns against consumption, but there are very few places where you can see a complicated organisation for this end at work. At Edinburgh, however, the Victoria Dispensary is the heart and centre of a scheme which comprises the Sanatorium or Royal Victoria Hospital, the Farm Colony, the Public Health Department, together with the Municipal Hospital for advanced cases. The dispensary is the sorting centre, and sends the patients who require institutional treatment to the Sanatorium if incipient cases, or to the Municipal Hospital, as the case may be. It has collected a vast amount of data, and is indeed a register and directory of consumption. To this the Medical Officer of Health is given access, in return for which the Municipality contribute £450 towards its working expenses every year. Prolonged supervision is carried on at the Farm Colony, but the register seems to show that the appearance on the scene of advanced cases is less frequent than it was a few years ago, when there was no home visiting. In the grounds of the hospital is a school for tuberculous children, which, if the School Board would only co-operate, might be made as effective educationally as it is hygienically to-day. The productivity of the Farm Colony seems amply to justify the plea for such work recently argued so practically in our articles on institutional outdoor life and administration, the last of which appears in this issue.

### DIFFICULTIES AT THE BROMPTON HOSPITAL.

While, owing to the Maharajah of Nepal's donation of £1,000, through Dr. Inman, the proposed extension of the Brompton Hospital clinical laboratories is being proceeded with, all work on the chapel for the patients at the Sanatorium has had to be given up temporarily for want of funds. For some reason or other, possibly the growing hold which institutions for the treatment of cancer have gained upon the public, this hospital has received few legacies of recent years, and has a bankers' loan of £10,000. In these circumstances the interim court of governors which was held last week was chiefly concerned with an appeal for money. To anyone who understands the pioneer work that is being done at Frimley, and its success, the claims of the Brompton Hospital for Consumption are very high indeed.

### PHYSICIANS, PATIENTS, AND SURGEONS.

There never was a time since the world began when the facilities for operative treatment, and the popularity of such treatment amongst all classes, were so nearly universal as they have become in the year 1911. We believe that the rush and fussiness of people in the present day has had much to do with the general acceptance without protest of a development in regard to treatment which will require continuous watching if it is to be kept within limits which it never ought to be permitted to exceed. Surgical treatment, as pursued to-day, involves continuous strain upon the surgeons and their assistants. The ordinary layman may come to realise this when it is stated that many an honorary surgeon attached to a great hospital has to perform four hundred or more major operations in the hospital each year, in addition to all those which fall to him in connection with the private practice upon which he has to depend for his maintenance. It is not within the scope of our present purpose to pursue this point further here, but the profession and the public may be reminded that there was a time in the history of medicine when surgical operations of importance were not permitted to be performed except in the presence of a physician, and with his consent. Personally, speaking with a full sense of responsibility and an intimate knowledge of the facts, we should say that it is highly probable that the aid of the physician, as representing the patient and his friends, may again become a most useful and well-recognised practice, where important operations are contemplated or may have to be performed.

### THE PROTECTIVE VALUE OF THE VOLUNTARY HOSPITAL.

It would be a national, nay, an international, calamity to destroy the voluntary hospitals and all that they represent by way of protection to all classes, and especially to those who are least able to care for themselves and their families when ill. In the voluntary hospital the claims of the patient, his needs and adequate protection are the first considerations with all who are responsible for its administration. No man or woman, however wealthy, can in fact enjoy all the safeguards and comforts and protective agencies which collectively make for rapid recovery and the minimum of suffering in the patient, which the occupant of a bed in the best administered voluntary hospital of to-day enjoys, free of all cost.

### WESTMINSTER HOSPITAL AND ITS CORONATION STAND.

At the time of the Coronation The Hospital published an illustration of the stand which, on its unique site, Westminster Hospital built to view their Majesties' arrival at the Abbey. We also gave some particulars as to the seating arrangements and as to the sum which the institution expected to raise by the sale of the seats. It is now announced that £2,000 has been applied to the endowment of two Coronation memorial beds, which will be named after the King and Queen respectively. Permission for this was obtained very shortly before their Majesties left for India.

### THE NEW WING AT THE STREATHAM HOME.

The British Home for Incurables at Streatham is about to build an additional wing, which will be christened, by permission, after Queen Alexandra.

The cost and furnishing are expected to reach £10,000, of which £5,000 has been raised already, while Mr. J. H. Mullens has given a further £5,000 to endow a room. At the half-yearly meeting over which Lord Strathcona presided, General Sir A. Montgomery-Moore proposed the vote of thanks to him, and was reminded that it was through his own father-in-law Lord Seaton, that Lord Strathcona entered the Hudson Bay Company.

### SEIZE THE CORRESPONDENCE COLUMNS OF THE PRESS!

Mr. George E. Priestman, chairman of the House Committee of the Bradford Royal Infirmary has set a capital example to all voluntary hospital committeemen by attacking certain wild statements on the Insurance Bill which have been made lately by his local paper, the *Yorkshire Observer*. A recent leading article contained the statement, says Mr. Priestman, that Mr. Lloyd George's fund "will relieve the burden on the hospitals." To this Mr. Priestman replies justly that "so far there is no provision in the Bill to bear out this assertion.' This letter continued by referring to the attitude taken up by the British Hospitals Association and to its fears that more cases will have to be dealt with at the very time when the subscriptions of employers and employed are growing less. The editorial comment of our contemporary on this letter, far from proving the existence of its alleged fund can only contend that "the existence of insurance benefits places the hospitals in a better position . . . to demand a proper arrangement before admitting patients not fairly entitled to charitable aid." How this is likely to relieve the burden on the hospitals does not seem clear. Mr. Priestman has set a capital example in insisting on the true position of the hospitals under the Bill being stated. The correspondence columns of the provincial press should be flooded with similar letters. Then members of Parliament would begin to realise the gravity of the hospitals' position. There have been far too few letters from hospital men, and the leader writers have been allowed to go their own way uncriticised. Mr. Priestman deserves high praise for leading what we hope will be a persistent and wide-spread newspaper attack.

### THE ADMINISTRATIVE CONTROL OF PHTHISIS.

Two years ago, owing to the number of tuberculous poor in Glasgow and to the pressure on the hospital accommodation, which includes the three large hospitals of Stobhill and the Eastern and Western District Hospitals, together with the extended Barnhill Poorhouse, the Local Government Board ordered an investigation into the administrative control of phthisis in Glasgow. A Blue-book is the result, in which three reports are included, by Dr. Dittmar, Medical Inspector to the Glasgow Parish Council; by Dr. Dewar, Medical Inspector to the Public Health Authority; and by Dr. Elizabeth M'Vail, Lady Inspector to the Govan district. The object of the inquiry was to determine the relative share of the local authority and of the parish councils in the control of phthisical cases, and whether all that might be done was done by means of their respective efforts. Numerous recommendations were made, among them the necessity for anti-tuberculous dispensaries, and for a nursing staff to undertake home visiting being insisted upon. Dr. Dewar also lays emphasis on the need for vesting the whole responsibility for the control of pulmonary phthisis in the local authority.

### THE ST. THOMAS'S GUILD AT THE MANSION HOUSE.

The fact that the new Lord Mayor, Sir Thomas Crosby, is a medical man lent particular interest to the meeting of the Ladies Guild of St. Thomas over which Miss Crosby presided at the Mansion House last week. The Guild provides clothing for destitute patients, and has solved a paradoxical situation which indeed it was created to remove. All patients who were recommended for drafting on to the convalescent homes were subject to a rule that they should come provided with fresh clothing. All the poorest patients were therefore debarred. The report was read by the honorary secretary Mrs. W. H. Battle.

### HOSPITAL EFFORT IN KENSINGTON.

The Kensington and Fulham General Hospital is continuing its campaign for efficiency and funds, which was inaugurated by Lord Claud Hamilton's chairmanship. A few days ago a pound day produced thirteen hundred articles of one pound weight, which is a very real tribute to the energy in attracting local support that the secretary, Mr. Louis McCausland, has exhibited. On Wednesday, December 13, a ball in aid of the hospital will be held at the Empress Rooms. Dancing will begin at 9.30 p.m. The price of tickets, including supper, will be 15s. 6d., and they may be obtained from Mr. J. E. Kitchingman, the treasurer, at the hospital, Earl's Court.

### DENTISTS AND ANÆSTHETICS AT TREATMENT CENTRES.

The provision of an anæsthetist at the various dental treatment centres under the care of the London County Council has often been urged in The Hospital, and we are now in a position to congratulate the Council on having carried out this much-needed improvement. In August the Council sanctioned the employment of an anæsthetist in connection with dental treatment at the Poplar Hospital, and it was then intimated that it was intended that this provision should be extended to all the other schemes. At its meeting on November 14, the Council renewed the agreements with the Deptford Children's Health Centre and the St. George's Dispensary, Blackfriars, for a further period, and at the same time an extra clause was inserted in the agreements to provide that an anæsthetist should be employed on one half-day a week at Deptford and one half-day a fortnight at Blackfriars. It was stated at that meeting that all the dentists concerned were in agreement that an anæsthetic was essential in difficult cases of extraction. The only remaining dental scheme which did not provide for the treatment of cases requiring an anæsthetic was the Wandsworth School Treatment Centre, and at its meeting on November 21 the County Council agreed that there also provision should be made for anæsthetics to be administered on one half-day each fortnight.

### PRESENTATION TO A LEICESTER SURGEON.

At a complimentary luncheon given by Sir Edward Wood, the Chairman of the Board of Governors of the Leicester Infirmary, to Mr. Claude Douglas, F.R.C.S., on the completion of twenty-five years' service as one of the Honorary Surgeons to the Infirmary, Sir Edward Wood, on behalf of the members of the Board and the hon. medical and surgical staff presented Mr. Douglas with an illuminated address and a gold watch and chain. The watch, on which were engraved the initials "C. D.," and the Douglas crest, bore the following inscription: "Presented to Mr. Claude Douglas, F.R.C.S., by members of the Board and his colleagues on the Hon. Medical and Surgical Staff of the Leicester Infirmary, on his retirement after twenty-five years of devoted service; November 15, 1911." It was agreed that a portrait be hung in the Institution, and that Mr. Douglas' name be recommended to the quarterly meeting of the Governors for election as Consulting Surgeon to the Institution.

### HOSPITAL CLERKSHIPS VACANT IN LONDON.

Among new institutional appointments which we had to hold over from last week is the transfer of Mr. Clapham from the Hampstead General Hospital, where he has held the office of chief clerk, to the Queen's Hospital for Children, Hackney Road, where he will act as assistant secretary. Those in search of hospital clerkships will note, therefore, vacancies at the Hampstead General Hospital and at the Royal Dental Hospital in Leicester Square. We understand that shorthand and typing are necessary for the latter appointment, which will carry a salary of £78 a year.

### HOSPITAL SECRETARIES' LEISURE HOURS.

Some secretaries have already informed us that they have for a long time considered the advisability of writing up their institutions in the way we often suggest. The Secretaries have the means of access to the archives and necessary documents, and are fully acquainted with the present state of their institutions; they are therefore admirably fitted to undertake such a work, and if they possess the necessary literary skill there will be leisure to accomplish it. But we would urge that in each case the history be made more complete and more generally instructive by incorporating in it special chapters dealing with the architecture, the medical and surgical work, and the administration of the institution. One of the best examples of the kind of book we have in mind lies before us at present, though it is the story of a hospital that has only existed for a few years. This is the finely-illustrated and well-written historical description of the Groningen Hospital by its able Superintendent, Dr. van Eysselsteyn, who joins to the knowledge of hospital administration which he possesses a terse, clear style and the ability to take a wide and comprehensive view of developments in hospital progress in other countries so far as they concern his own institution. Dr. van Eysselsteyn's book is undoubtedly one of the most interesting monographs on hospital construction which has been printed during recent years, and a glance at it should encourage hospital secretaries to attempt something similar for their own institutions. It is the best way of spending their little leisure.

### THE SHEFFIELD EXTENSION.

The recent completion of the new wing which has been added to the Sheffield Infirmary practically fulfils the scheme that was outlined in 1893. But a recent announcement reminds the public that the new block cannot be occupied fully until a new home has been provided for the nursing and domestic staffs. A site for this, we understand, has been bought in Devonshire Street. At the moment, however, as much thought is being given to the noise created by the trams which pass one side of the hospital as to anything else. Now that the neighbouring street widening scheme has taken shape, and that the trams have been fitted with washers it is hoped that this nuisance will be done away with before the new block is ready and the new home equipped.

### A HOSPITAL GOVERNOR'S FUNCTIONS.

In the general education of the public in hospital matters which the voluntary system has brought about, and on which indeed it depends, the methods by which the great hospitals are governed and the functions of the governors are not nearly as well known as they should be. Yet the division of labour which the method of hospital committees is designed to realise is a subject not without interest. At St. Bartholomew's, for instance, to take a most prominent case, the visiting of the hospital and of its convalescent home, the raising and expending of money, even the purchase and control of instruments and drugs, is each in the hand of a separate committee. Though it may not be easy to get the public, or the governors, to attend routine meetings, there is no doubt that the public likes to be introduced to, and to understand, the machinery of management. We hope this will be done well in the promised history of St. Bartholomew's, at least in the last volume. Was it not out of such facts as these that Mr. E. W. Morris made such good propaganda in his book?

### CONTROL OF CHARITIES IN AUSTRALIA.

In reply to a deputation (writes an Australian institutional correspondent) the Victorian State Treasurer (Mr. Watt) stated that he intended to bring in a Bill for the better control of charities; that under it a central board would be created, and that the colony for epileptics, at present not under Government control, and the Infectious Diseases Hospital, partly municipal, will be placed on the same footing as others. " All that is best in the present system will be retained; all that is worst, eliminated." Until this millennial result is attained, he advised everybody not to worry about central offices. About the same date in Sydney, the Acting Chief Secretary (Mr. Flowers) discovered that " the present financial control and management of our hospitals are wrong." As outlined, various points have recently cropped up, which show the need for such control. The differences between the Managers of the Alfred Hospital and the Victorian State Treasurer, after nearly leading to a rupture, are in process of adjustment. The appointment of a clinical pathologist, who was wanted, Mr. Watt first understood, as a teacher for the students, until one of the deputation explained that he was really needed for the making of vaccines, is likely to be proceeded with. Mr. Watt did some hard hitting—on the manager, doctors, and secretary alike.

### "LONG STAY" OF PATIENTS AT MELBOURNE.

But the atmosphere seems clearer since the interview, although some matters are still unexplained, especially the long stay of patients—the longest of any Melbourne hospital. Agreement on the subject of hospital supplies has also just been arrived at between the State Treasurer and the authorities of the Metropolitan hospitals. Representatives waited upon him to report progress. Conferences had been held with the result that it had been proposed to appoint an Advisory Board of representatives of each of the Metropolitan hospitals, to meet regularly and supervise generally the purchase of all requirements of the various institutions, whether by contract, or the open buying system, or partly by one system or the other, as might seem best. One speaker remarked that the trenchant manner in which the Treasurer had dealt with hospital supplies might prove a blessing in disguise. Mr. Watt expressed his pleasure in accepting these recommendations—the experiment to have a trial of two years. One woman, at least, will be on this Advisory Board, from the Queen Victoria Hospital.

### CHAIRMAN OF THE JESSOP HOSPITAL RESIGNS

Mr. C. D. Pettinger, whose bad eyesight has interfered for some time with his direction of the Jessop Hospital for Women, Sheffield, has now sent in his resignation. Three years ago, in March 1908, Mr. Pettinger became chairman of the Board, on which he had had a seat for many years. Since his election to the chair the heating of the hospital has been renovated by the installation of a new system. Mr. Pettinger, who will remain a member of the Board, will be succeeded for the time being by Mr. Charles Yeomans, who became a member of the Board in February 1905, at the same time as Mr. Pettinger. Mr. Yeomans has been a member of the Board of Management of the Retreat at York for many years.

### TREATMENT OF CHILDREN ON ADMISSION TO HOSPITAL.

Dr. Northrup, in a recent number of the *Archives of Pediatrics*, describes the methods of procedure with regard to children at the hospital with which he is connected. All children are first admitted to a ward called the " observation ward." Dr. Northrup himself visits this ward every day in order to decide whether there would be any risk in admitting the cases to an ordinary ward. Cultures are made from the nose and throat and smears taken from the vagina before the case is considered eligible for admission. When admitted to the ward the child is still for a time " a suspect." Around " suspects " are placed screens of latticed wire on strong metal mountings to keep the children who are up—" the runabouts "—from visiting within the radius of coughing and sneezing. Remarks are also made upon methods of bathing. The bath-room is thoroughly heated, so that " every surface, every fabric, the slabs, and the water are all agreeably warm." Lastly, the children are bathed by means of a spray. The old-time bath is considered to be thoroughly out of date. It is said " to fill a bath-tub, secure the correct temperature, bathe the baby, empty the bath-tub, clean it, disinfect it, refill it, and secure the proper temperature requires time and a conscientious worker." The water used for washing the babies flows from a tank in which the water is heated to 103° F. and flows out at about 100° F.

### THE SCIENTIFIC BATH.

The baby is washed on a warmed marble slab, and the towels for drying the baby lie on another warmed slab. After the bath fresh clothing is brought dry and warm from a hot closet. The towels used for drying the baby are boiled and again used for the same child the following day. Another device used in the same hospital is the electric crib. Dr. Northrup remarks that " a baby with cold feet cannot digest, and a poorly nourished baby must have artificial heat until it can furnish its own. Hot-water bottles are expensive, troublesome, and perilous. In using many hot bottles for an extended time accidents of burning are sure to occur." The electric crib is designed to obviate the disadvantages of the hot bottle. The crib consists of a shallow asbestos box containing an electric heater. Above the heater is an asbestos trough in which is placed the bassinet. No more electricity is said to be required for the heater than is supplied to an ordinary illuminating bulb.

### THE PHARMACIST'S DIFFICULTIES WITH OUT-PATIENTS.

The most important point which strikes a pharmacist who has been appointed to the dispensing department of a large general hospital is the methods employed in handling dangerous poisons. The experience of the pharmacist who has been employed previously in the other branches of pharmacy has been strictly confined by the two schedules of poisons named in the Poisons and Pharmacy Acts of 1868 and 1908. The first required him to know the persons who desire to obtain these drugs, their addresses, and the purpose for which the drug is to be used. By the second the duties he had to perform were principally those regarding labelling. By these Acts of Parliament medical men are especially exempted from the provisions and regulations, and when a pharmacist enters hospital service he is under the nominal control of the medical superintendent. The provisions of the Poisons and Pharmacy Acts do not legally apply. Nevertheless hospital pharmacists do take great trouble to prevent dangerous drugs from being improperly employed and in many cases it is very difficult to make the patients (in the out-patient department) understand that it is necessary to adopt special precautions. Sometimes it seems impossible to make them understand that most cases of poisoning are due to accidents. There seems to be no doubt that the hospital pharmacists are doing good work in educating the poorer class of people of the dangers in the careless handling of poisonous drugs. Probably from the fact of handling such quantities of drugs these pharmacists (at any rate in the larger institutions) are usually working in advance of the times with regard to the precautions employed, and it is certain that they are greatly in advance of the legal conditions laid down by law. For instance, we know at least one institution in which it has been the rule for several years to take special precautions in the supply of liquid ammonia, yet the proper regulations and distinctive bottle will only come into force on February 1, 1912.

### CERTIFICATED DISPENSERS UNDER THE INSURANCE BILL.

Among the latest steps that have been taken to safeguard the interests of those holding the dispenser's certificate was the reception last week by a member of the Government of Mr. Albert Howell, Honorary Secretary of the Association of Certificated Dispensers, and of Mr. F. E. Trayner. The distinguished gentleman was, it seems, a little vague and non-committal, but the dispensers at least will feel that their claims have been stated authoritatively to those who can influence legislation.

### A NEW BOOK FOR DISPENSERS.

"The British Pharmaceutical Codex" is the name of a new book issued by the authority of the Council of the Pharmaceutical Council of Great Britain. Its object is to provide standards for preparations which are not in the "British Pharmacopœia," for the use of prescribers and dispensers. The moment of publication aptly coincides with the threatened passing of the separation of prescribing from dispensing, which forms part of the Insurance Bill. The reason why so many preparations are not contained in the "B. P." is, of course, that that official work is thirteen years old. Indeed, the index to the new volume contains some fourteen thousand items which are all drugs or medicines in common use throughout the Empire.

### THIS WEEK'S DRUG MARKET.

Few important changes in the value of drugs have taken place during the week and on the whole business has been quieter. Makers of chloroform have rearranged their quotations, which are 4d. per lb. below former prices, the reduction being mainly due to competition. A large business has been done in quinine, mainly on the part of speculators and the price in consequence tends higher although no material advance is expected; some years ago quinine was a favourite object of speculation but the enormous supplies of cinchona bark which now come on to the market have reduced the probabilities of fluctuations in value very considerably, with the result that most of the business done nowadays is in response to the legitimate demand for medicinal purposes. The present price may be considered very low and buyers would not go far wrong in replenishing their stocks. Opium maintains its high value and an advance in the price of codeine is expected. It is not unlikely that a small advance in the price of camphor may take place; in any case camphor would appear to be a safe article to buy at present quotations. Balsam copaiba is tending slightly higher in value. Cod-liver oil is in comparatively small demand considering the season of the year; buyers are no doubt restricting their purchases in the expectation of lower prices. At the recent public sale of drugs in Mincing Lane, slightly lower prices were paid for ipecacuanha and jalap.

